# Portable Brain-Computer Interface for the Intensive Care Unit Patient Communication Using Subject-Dependent SSVEP Identification

**DOI:** 10.1155/2018/9796238

**Published:** 2018-02-05

**Authors:** Omid Dehzangi, Muhamed Farooq

**Affiliations:** Computer and Information Science Department, University of Michigan-Dearborn, 4901 Evergreen Rd., CIS 112, Dearborn, MI, USA

## Abstract

A major predicament for Intensive Care Unit (ICU) patients is inconsistent and ineffective communication means. Patients rated most communication sessions as difficult and unsuccessful. This, in turn, can cause distress, unrecognized pain, anxiety, and fear. As such, we designed a portable BCI system for ICU communications (BCI4ICU) optimized to operate effectively in an ICU environment. The system utilizes a wearable EEG cap coupled with an Android app designed on a mobile device that serves as visual stimuli and data processing module. Furthermore, to overcome the challenges that BCI systems face today in real-world scenarios, we propose a novel subject-specific Gaussian Mixture Model- (GMM-) based training and adaptation algorithm. First, we incorporate subject-specific information in the training phase of the SSVEP identification model using GMM-based training and adaptation. We evaluate subject-specific models against other subjects. Subsequently, from the GMM discriminative scores, we generate the transformed vectors, which are passed to our predictive model. Finally, the adapted mixture mean scores of the subject-specific GMMs are utilized to generate the high-dimensional supervectors. Our experimental results demonstrate that the proposed system achieved 98.7% average identification accuracy, which is promising in order to provide effective and consistent communication for patients in the intensive care.

## 1. Introduction

A major problem for mechanically ventilated patients in the Intensive Care Unit (ICU) is their inability to consistently and effectively communicate their most fundamental physical needs. Patients rate about 40% of communication sessions as difficult and more than a third of communications about pain as unsuccessful [1, 2]. Nurses initiate about 86% of all communication exchanges as it is typically very difficult for a voiceless patient in the intensive care to initiate communication. Patients in the ICU therefore commonly suffer unrecognized pain and discomfort and feelings of loss of control and insecurity, depersonalization, anxiety, sleep disturbances, fear, and frustration [1, 3]. Caregivers also frequently report feeling anxious and frustrated in not being able to adequately assess the needs of their patients [4]. This inability to communicate effectively can lead to the inappropriate use of sedatives and prolongation of time spent on the ventilator [5], which may then lead to increased ICU length of stay and costs [3]. Furthermore, the inability to communicate with caregivers hampers the ability of critically ill patients to be active participants in their treatment and in decision-making, including decisions to withdraw or withhold life-sustaining treatment.

Mechanically ventilated patients in the intensive care are voiceless and unable to communicate their needs verbally [6] and their inability to communicate adequately can lead to fear, panic, and insecurity [7]. The primary means of communication for these patients is the use of nonvocal techniques, such as lip reading and gestures [8, 9], which are often inadequate for effective communication with family and ICU staff [10–12]. The use of picture boards with icons representing common patient needs and complaints (pain, fear, hot/cold, thirst, bedpan, etc.) has been shown to improve nurse-patient communication for patients in the postoperative period on the ventilator [13]. These picture boards are widely available in most ICUs and are the closest approach to a current standard for communication with voiceless mechanically ventilated patient, for the purposes of addressing fundamental physical and emotional needs. Recent pilot studies have described the use of computer-assisted communication using touch sensitive screens, eye blink detectors, and gaze trackers to enable communication in the ICU department [14, 15]. The majority of patients and hospital staff surveyed in these studies indicated that the use of a computer-assisted communication device improved their ability to respond to patient needs and address patient comfort. However, touch sensitive screens may not be suitable for the majority of patients in the intensive care with weakness and restriction in motor ability. About 25% of patients requiring mechanical ventilation in ICUs may have significant weakness from critical illness, Neuropathy and Myopathy, limiting their ability to use their hands to select the appropriate icon on a picture board or touch screen pad [16]. There are several patient populations, however, for whom the use of a picture board or touch screen is impossible, including patients with high spinal cord injury, advanced ALS, and brainstem stroke, who are voiceless, but also typically have no useful motor function of their limbs. These patients are locked-in, to varying degrees, awake, and alert but with no control of bodily functions or ability to articulate and communicate using standard forms of communication [17]. Eye blink detectors and gaze trackers that are based on eye movement activities and muscle movements might or might not be feasible for the aforementioned patient population. Furthermore, those technologies have privacy issues, due to patient video streaming requirement, are sensitive to illumination and viewing angle, and require the eyes to be wide open [18]. The use of Brain-Computer Interface (BCI) devices to facilitate communication for voiceless patients has recently generated an interest. A BCI translates deliberate, involuntary modulation of cerebral electrical activity, typically recorded by electroencephalography (EEG) into computer commands. BCI technology can directly interpret and relate the brain patterns into the control commands and can bypass all other body functions to communicate the intent of a patient. BCI devices in the ICU are mostly used for continuous patient monitoring [19–22]. A variety of BCI devices have been used to permit patients with advanced Amyotrophic Lateral Sclerosis (ALS), high spinal cord injury, and brainstem stroke with the locked-in-syndrome, who have no voluntary use of their limbs, to communicate to varying degrees [23–27]. These devices have typically been evaluated in the rehabilitation setting, following the period of an acute medical illness, or at home. No study has evaluated the use of a BCI device to assist with communication of the typical physical and emotional needs/complaints of the critically ill. This is significant, not only for patients with spinal cord injury and stroke, most of whom are initially admitted to an ICU, but to the potentially large number of patients in the intensive care who cannot use a picture board or other finger contact systems because of a critical Neuropathy/Myopathy illness or an acute brain injury that causes weakness. Our objective is to create an end-to-end steady-state visual evoked potential- (SSVEP-) based wireless BCI system to facilitate communication with intubated patients in the intensive care [28–30]. The portable BCI device of interest in this study functions through visual attention to illustrative icons displayed on an Android tablet screen. The icon, which depicts a common need of a patient in the intensive care, such as the need for repositioning, common patient complaint, or pain, is displayed as a symbol flickering at a specific frequency, which then drives a corresponding frequency of an electrical EEG signal, permitting the BCI device to identify the specific item that the patient is focusing on [31, 32]. The patient can, thereby, communicate by looking at a specific item depicting their need or complaint. This wearable device is entirely noninvasive and without significant risk to patients, functioning only to record and translate EEG signals. The device communicates wirelessly with the user interface (UI) on a tablet that is present in every patient room. The proposed prototype provides a user interface that includes basic functions that are typically used in communication between nurses and patients in the ICU, with the capability of being customized for each patient.

Generally, ICU needs are often required to be communicated quickly and easily, rather than through spelling of words and sentences. Therefore, we propose a system of rapid and reliable communication of typical ICU needs. The proposed BCI4ICU system utilizes a wireless EEG cap. The system is designed and optimized to perform in real-world scenarios involving patients in the intensive care. It includes three major modules: (1) our custom designed Android-based wireless data acquisition and processing platform app, (2) the Android-based openGL paradigm stimuli generation module on the mobile device, and (3) the novel GMM-based signal processing and SSVEP identification algorithm to fit the requirements of the ICU application.

Several efforts investigated improving current BCI systems by overcoming their well-known drawbacks, such as lack of reliability, user accessibility, and low information transfer rates utilizing hybrid BCI systems. Hybrid BCIs entail combining two different systems either sequentially or simultaneously [33]. In simultaneous hybrid BCIs, both systems operate concurrently and in parallel, whereas, in sequential hybrid BCIs, the output of one systems is employed as an input for the other system [33]. One such effort was conducted by Hong and Khan, where they investigated designing a noninvasive hybrid BCI utilizing EEG signals in conjunction with other brain/nonbrain modalities, such as functional near infrared spectroscopy (fNIRS), electrooculography (EOG), and electromyography (EMG) [34]. The aim of the study was to reduce the signal detection time, increase the number of control commands by combining SSVEP with P300, and finally enhance the classification accuracy by combining cognitive tasks with motor imagination/movement tasks.

Numerous other studies probed into enhancing current BCI systems by employing Gaussian Mixture Models (GMMs) for EEG signal analysis. Prabhakar and Rajaguru investigated utilizing approximate entropy as a feature extraction method followed by Sparse Representation Classifier (SRC) and GMMs to classify epilepsy risk levels from the patients EEG signals [35]. Wang et al. suggested a signal detection approach for BCI technologies [36]. In their analysis, signal detection was implemented by training GMMs on the resting brain activity so as to detect any imagined and/or real movement. As such, their experimental results demonstrate the feasibility of this approach. Zhang et al. examined the improvement of the classical Common Spatial Pattern (CSP) coupled with support vector machine (SVM). Their proposed method entails establishing a number of mixture models in the CSP feature subspaces utilizing a GMM-based feature learning algorithm in conjunction with a probabilistic model in order to depict the EEG distribution features of stroke patients and finally classify their EEG signals [37].

In this paper, we propose a novel signal processing solution that encompasses extracting the discriminative and complementary information of Canonical Correlation Analysis (CCA) and Power Spectral Density Analysis (PSDA) and combining the extracted information at the score level to generate discriminative fusion spaces. Subsequently, we derive the subject-specific GMMs from the generated fusion spaces and then perform our discriminative analysis.

## 2. Specific Technical Challenges

- Calibration: to acquire subject-specific information, BCI systems generally require a calibration step at the beginning of each recording session. Such necessity can be cumbersome for patients in the intensive care.* As such, our proposed system design begins responding to patient's communications using a baseline model while it captures and integrates the subject-specific information during the feature extraction and model training phases to improve the correct response rate*.
- SSVEP paradigm generation accuracy: SSVEP technology relies largely on a set of target objects, which serve as the visual stimuli, flickering on a screen with fixed frequencies. The precision of the SSVEP paradigm generation is determined by the hardware specifications of the machine generating it. To accommodate portability of the system, the paradigm is operated on an Android tablet with an insufficient screen refresh rate. Furthermore, due to the intermittent operating system interruptions, the visual stimuli might suffer from significant imprecision in the flickering frequency of the target objects in fractions of a second.* Thus, we employ a feature extraction framework in order to mitigate the effect of the imprecise SSVEP paradigm generation and to take the introduced uncertainty into account in our decision-making process*.
- Asynchronous communication in the ICU: communication paucity can cause distress to doctors/nurses and patients. The ideal situation is that the communication is initiated by the patient on demand and completed while the nurse is reaching out.* Therefore, our system provides effective communication to the patients based on their needs/complaints utilizing the divide-and-conquer approach comprised of a two-stage system design. First, the system detects when the patient needs to initiate communication. Second, find out the specific patient needs to communicate effectively*.
- Number of target stimuli limitation: in addition to the insufficient screen refresh rate, another challenge for BCI systems in the ICU is the convenient number of target objects rendered on the visual stimuli to avoid interference with the patients visual perception.* Therefore, based on our feedback from the NICU doctors and nurses, we designed an optimized and more sophisticated stimuli flow that will communicate the patients' needs effectively utilizing the target frequencies that the patient is most responsive to*.
- EEG nonstationarity: EEG signals demonstrate significant variation between sessions and between subjects. This is primarily due to changes in the biological conditions of subjects, such as fatigue and emotional/mental state. Furthermore, electrode-scalp locations and the quality of the acquired signals are also causing factors.* As such, we capture the variation between different subjects and employ it to generate an improved subject-specific identification model using GMM training and adaptation*.

## 3. BCI4ICU System Architecture

**Figure 1:**
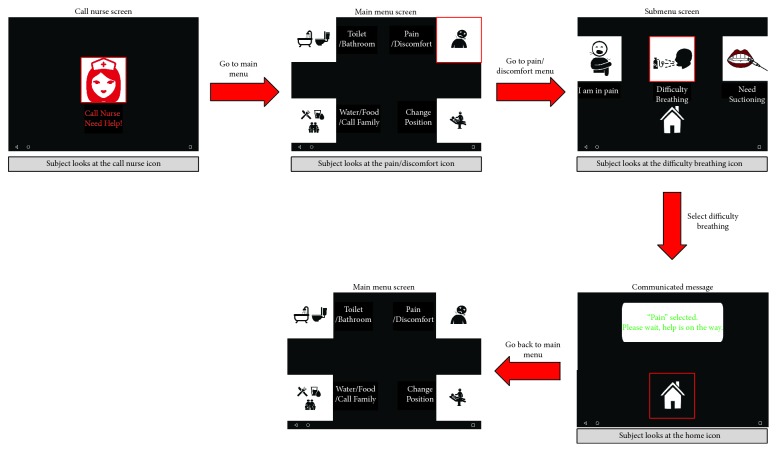
System's call cycle example.

**Figure 2:**
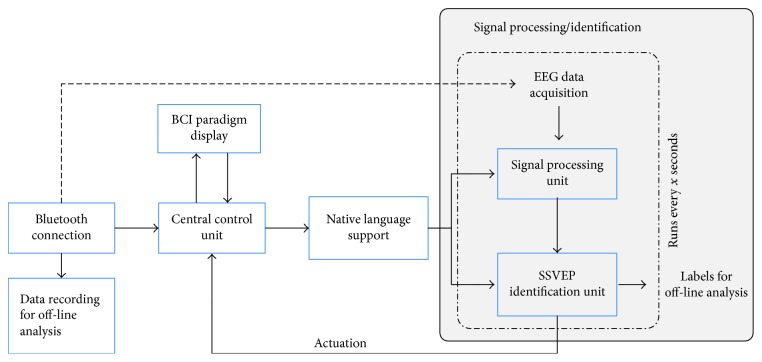
JNI-Android wireless data acquisition and processing platform.

The proposed BCI4ICU system is designed to operate in an ICU environment to allow patients to communicate their needs effectively. The system architecture is comprised of 3 modules:

1. * JNI-Android wireless data acquisition and processing platform*: the systems architecture that controls real-time SSVEP paradigm generation, EEG data acquisition, signal processing and modeling modules is depicted in Figure 2.
2. * Android-based OpenGL stimuli paradigm generation module*: our end-to-end closed-loop platform utilizes SSVEP-based BCI, which requires visual stimulation. The visual stimulation is provided on an Android tablet that displays a call the nurses screen and then renders 4 different icons, each of which indicates a specific message. After focusing on a specific icon, the patient is presented with a submenu from which he/she can select a command to communicate with the medical staff (see Figure 1).
3. * The novel GMM-based signal processing and SSVEP identification module:* in order to fit the technical requirements of the ICU application, we propose a subject-specific GMM-based SSVEP identification solution. We present the details of the proposed signal processing module in Section 4.
4. Figure 2 illustrates the overall process flow of the BCI4ICU system. The SSVEP paradigm generation module runs concurrently with the data acquisition module to acquire the EEG data. Subsequently, the signal processing module runs every 10 seconds to process the EEG data and feeds it to the predictive model to obtain the SSVEP identification accuracies.

## 4. Proposed Signal Processing Methodology

### 4.1. Data Collection and Signal Processing

Ten healthy subjects participated in our experiment. Moreover, the data collection process in this work was approved by University of Michigan Institutional Review Board under the study ID: HUM00100788. The experiment was conducted in a lab environment where subjects were seated on a comfortable chair 20 inches from a 10.2-inch Liquid Crystal Display (LCD) Android tablet screen with a 2560 × 1800 screen resolution.

**Figure 3:**
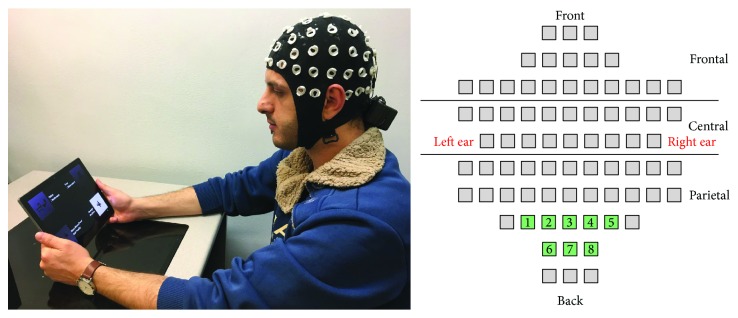
Portable BCI setup and channel locations.

The Cognionics EEG device was used to collect EEG data from 8 channels with a sampling rate of 250 Hz. Electrodes were placed on the occipital and parietal regions of the brain since it has been demonstrated that these areas contribute significantly to SSVEP identification [38].  Figure 3 illustrates the setup and electrode placements. Once the data is imported into MATLAB, we apply a 60 Hz notch filter and a 5th-order Butterworth bandpass filter. The filtered data is then passed to CCA to calculate the CCA coefficients and to PSDA to generate the signal's power scores. Unlike CCA, the challenge with PSDA was that because the EEG data was collected from 8 channels, PSDA generated an 8-dimensional power scores matrix. Therefore, we heuristically (1st-max-2nd-max) find out which channel responded the best for each subject to select for partitioning.

### 4.2. Task and SSVEP Paradigm Generation

**Figure 4:**
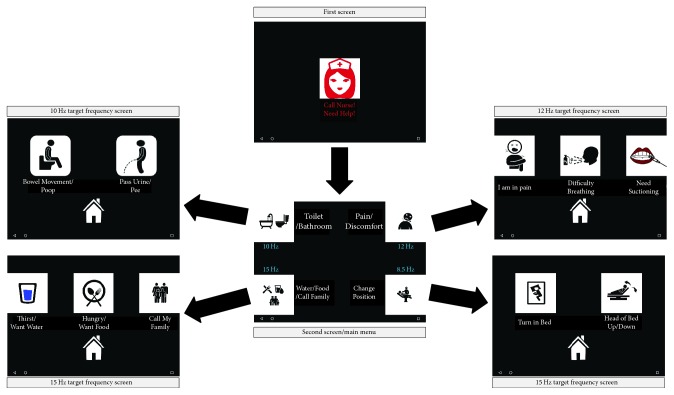
Experimental paradigm of the training session.

Four different icons were rendered on each corner of the Android tablet screen. Each icon was 600 pixels in size and flickered with a specific target frequency. Target frequencies were 10 Hz, 12 Hz, 15 Hz, and 8.5 Hz, respectively.  Figure 4 demonstrates the experimental paradigm of the data collection session. First, subjects focus on the call nurse icon to transition to the main menu screen, where the 4 target frequencies are rendered. Subsequently, subjects gaze at the first target frequency icon (i.e., the 10 Hz target frequency represented by the Toilet/Bathroom icon). If they transition to the corresponding 10 Hz target frequency screen, we consider that a successful call with the label (1); otherwise, we consider it an unsuccessful call with a label of (0). As such, we proceed to record more data until we obtain 10 successful calls per each target frequency. Most subjects required more than 10 trials to record the 10 successful calls for each target frequency (~70 trails per subject) generating a sufficient dataset size to evaluate the generalization capabilities of the proposed method.

### 4.3. Score Space Partitioning

**Figure 5:**
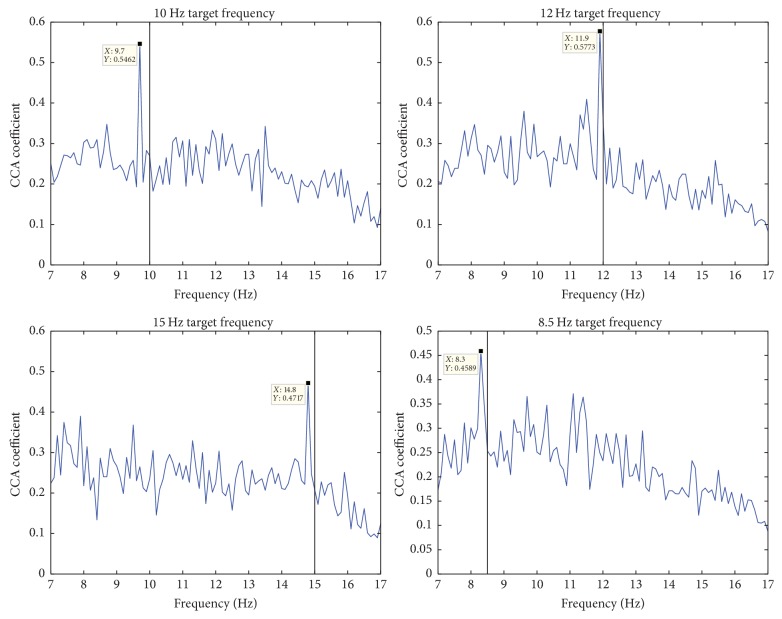
The effect of imprecise SSVEP paradigm generation.

Despite the feasibility and portability of the BCI4ICU system, one major challenge is the inaccurate SSVEP paradigm generation. This is mainly due to the insufficient screen refresh rate of the tablet and the recurrent interruptions by the Android operating system (See Figure 5 and Table 1). Table 1 illustrates the required time to display the target object on the screen during 4 subsequent epochs of a 10-second segment. The second column shows the desired timing for each of the target frequencies. In some epochs, the divergence is considerable and, as such, subject SSVEP responses are affected accordingly.

**Figure 6:**
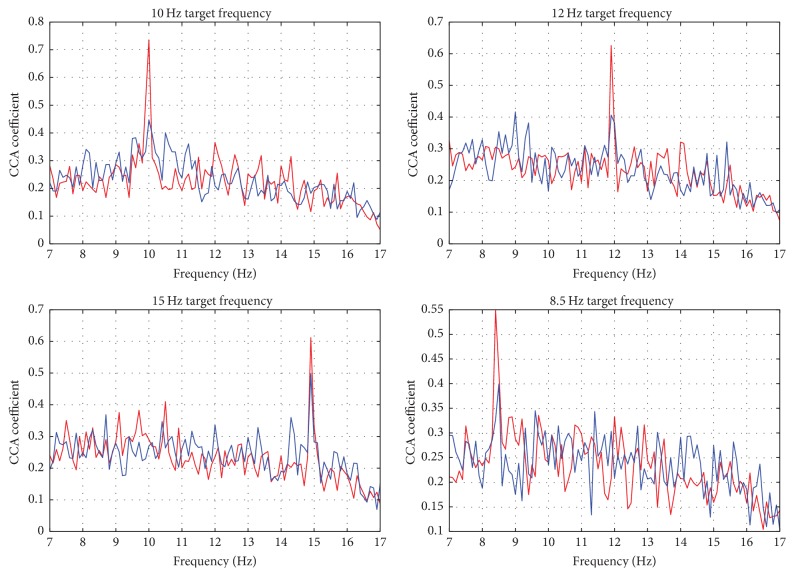
CCA responses of two different subjects.

In common BCI investigations, target frequency identification from SSVEP responses involved focusing only on target frequencies. It is not particularly an issue because the CRT monitors in most labs are high precision stimuli generators that do not introduce imprecise SSVEP paradigm generation. From Figure 5, we can observe that the peaks might not occur precisely on the intended target frequencies. This is because in various fractions of a second the frequency of the flickering stimuli is deviating from its original and desired value. Furthermore, we hypothesize that there is subject-specific information in the SSVEP responses over the whole frequency spectrum.  Figure 6 shows the variation in the output score space of CCA for two subjects across all 4 target frequencies.

**Figure 7:**
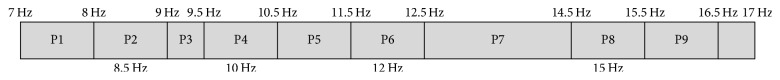
Score space partitioning of CCA and PSDA spaces.

As such, to mitigate the effect of the insufficient refresh rate and alleviate the ramifications of subject variation, we leverage the discriminative and complementary information of CCA and PSDA by partitioning their score spaces into 9 nonoverlapping partitions spanning the whole frequency range from 7 Hz as the minimum frequency to 17 Hz as the maximum frequency (See Figure 7).

The underlying concept behind the design of the partitioning scheme is to ensure that each target frequency is contained within a partition to capture the subject-specific information on and/or near the target frequencies and to evaluate the discriminative capabilities of the extracted measures from each partition to generate a discriminative score space that enhances the subsequent subject-specific models in the SSVEP identification task.

### 4.4. Partition-Based Feature Extraction

Feature extraction is a process from which informative measures are derived with the aim of facilitating the subsequent generalization steps. As such, we extracted 4 features from CCA's score space, namely, power, mean, standard deviation, and entropy. On the other hand, we extracted only two features from PSDA's score space, mean and standard deviation. Power was not extracted as a feature since PSDA inherently generates power scores of the signal. Additionally, extracting entropy was hampered by the insubstantiality of PSDA's power scores and was therefore omitted. Subsequently, we concatenated the extracted features to generate a 54-dimensional fusion space (4 features × 9 partitions from CCA and 2 features × 9 partitions from PSDA).

### 4.5. GMM-Based Modeling and Classification

As we mentioned in Section 2, BCI systems require a calibration stage before use. This is primarily due to the fact that patients respond to the generated SSVEP paradigms subjectively. Moreover, predictive models and/or subject-independent classifiers have no prior knowledge about the subject who is generating the SSVEP responses. To further elucidate, from Figure 6, we observe how subjective the CCA responses of 2 different subjects are in terms of the amplitude and the location of the peaks across the various target frequencies. Additionally, we also note the subjective responses of the nontarget frequencies which we hypothesize are the result of internal subjective responses to specific frequencies and/or external factors, for instance, the effect of visual interference due to multiple target stimuli. Therefore, to overcome this challenge, we suggest a GMM-based discriminative transformation and classification approach to capture and incorporate the discriminative subject-specific information.

*(1) Modeling Utilizing GMMs*. A Gaussian Mixture Model comprises a limited mixture of multivariate Gaussian components. Given a feature vector **x**, a GMM, denoted by *λ*, models a distribution as follows:

(1) $$\begin{array}{c} p\left(\;x\;|\;\lambda \;\right)=\overset{M}{\underset{m=1}{\sum }}\xi_{m}g_{m}\left(\;x\;\right), \end{array}$$

where *ξ*_*m*_ indicates the weight of the *m*th component and *g*_*m*_(**x**) represents the *d*-variate Gaussian function with its mean vector, *μ*_*m*_, and covariance matrix, Σ_*m*_,

(2) gmx=Nx ∣ μm,Σm=12πd/2Σm1/2exp⁡−12x−μmt∑m−1x−μm,

where *d* represents the dimension of the input feature vector, while the covariance matrices Σ_*m*_ are usually diagonal due to the fact that estimating the full-covariance GMM parameters requires more training samples and its computational cost is more significant, whereas the GMM density is multimodal and consists of a linear combination of Gaussian basis function, *g*_*m*_(**x**), capable of approximating random and continuous density functions [39]. A Gaussian Mixture Model can be viewed as an amalgamation of a simple Bayesian discriminant that utilizes 1 Gaussian density and a vector quantization codebook, which can model arbitrary probability densities [39].

GMM training involves the estimation of the GMM parameters represented by the weights, *ξ*_*m*_, the mean vectors, *μ*_*m*_, and the covariance matrices, Σ_*m*_, of each individual Gaussian density *g*_*m*_(·) employing part of the available training data, while determining the GMM parameters is achieved by estimating the maximum likelihood, which is calculated by the iterative expectation-maximization (EM) algorithm.

**Figure 8:**
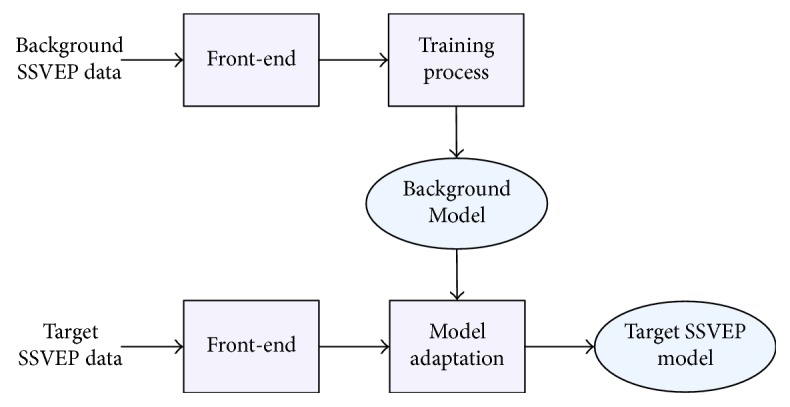
Training the background SSVEP model and subject adaptation.

*(2) GMM-Based Subject-Dependent SSVEP Identification*. SSVEP identification indicates automatically distinguishing a target object flickering with a specific target frequency that a subject is focusing on utilizing the information embedded within the SSVEP response.  Figure 8 depicts training a GMM-based SSVEP identification system. The feature vectors that carry subject-specific information are extracted by the front-end module. Additionally, the statistical redundancies are alleviated by a partition-based CCA and PSDA feature extraction. First, a collection of background SSVEPs of various subjects is employed to train a universal background model (UBM). Subsequently, to generate subject-specific GMM models, each GMM model is adapted from the background model instead of training from scratch. This is accomplished by using a collection of the corresponding SSVEP segments of each subject. Hence, effective estimation of the GMM parameters can be achieved even with a small number of data samples per each subject. On the other hand, subject adaptation can be accomplished by adapting all the parameters from the background model, or some of them utilizing maximum a posteriori (MAP).

Assume the enrollment segment for subject *ϕ*, **X**_*ϕ*_ = {**x**_1_, **x**_2_,…, **x**_*T*_*ϕ*__}, where *T*_*ϕ*_ represents the segments number in the SSVEP segment and *μ*_*i*_ represents the *i*th mean vector of the UBM, and *μ*_*i*_^*ϕ*^ is the *i*th mean vector of the adapted model for subject *ϕ*, determined by utilizing the maximum a posteriori (MAP) as the weighted sum of subject *ϕ*'s data, while the UBM means

(3) $$\begin{array}{c} \mu_{i}^{\phi }=\alpha_{i}x_{i}^{\phi }+\left(\;1-\alpha_{i}\;\right)\mu_{i}, \end{array}$$

where

(4) αi=lili+η,xiϕ=1li∑t=1Tϕpi ∣ xtxt.li=∑t=1Tϕpi ∣ xt,pi ∣ xt=ξiNxt ∣ μi,ΣiΣm=1MξmNxt ∣ μm,Σm

MAP adaptation is employed in order to obtain subject-specific GMMs from the UBM. The effect of the target subject data **X**_*ϕ*_ on the GMM model is controlled by the *η* parameter. In the identification stage, an SSVEP segment **X**_test_ = {**x**_1_, **x**_2_,…, **x**_*T*_test__} is utilized to identify the subject, whereas, for a group of S subjects, who are represented by the subject models, {*λ*_1_, *λ*_2_,…, *λ*_*S*_}, the aim is to obtain the subject model with the maximum log-likelihood, given the sequence of the input SSVEP segment, **X**_test_. Assuming that observations are independent from each other, the decision rule is

(5) $$\begin{array}{c} \hat{s}=arg\;\underset{1\leq s\leq S}{max}\overset{T_{test}}{\underset{t=1}{\sum }}\log p\left(\;x_{t}\;|\;\lambda_{s}\;\right). \end{array}$$

A subject is identified as $\hat{s}$, which corresponds to the model that increases the sum of the log-likelihood scores over the complete SSVEP segment. We consider the described GMM-based system as the baseline system in this study.

### 4.6. GMM Likelihood Vectors and Supervectors

**Figure 9:**
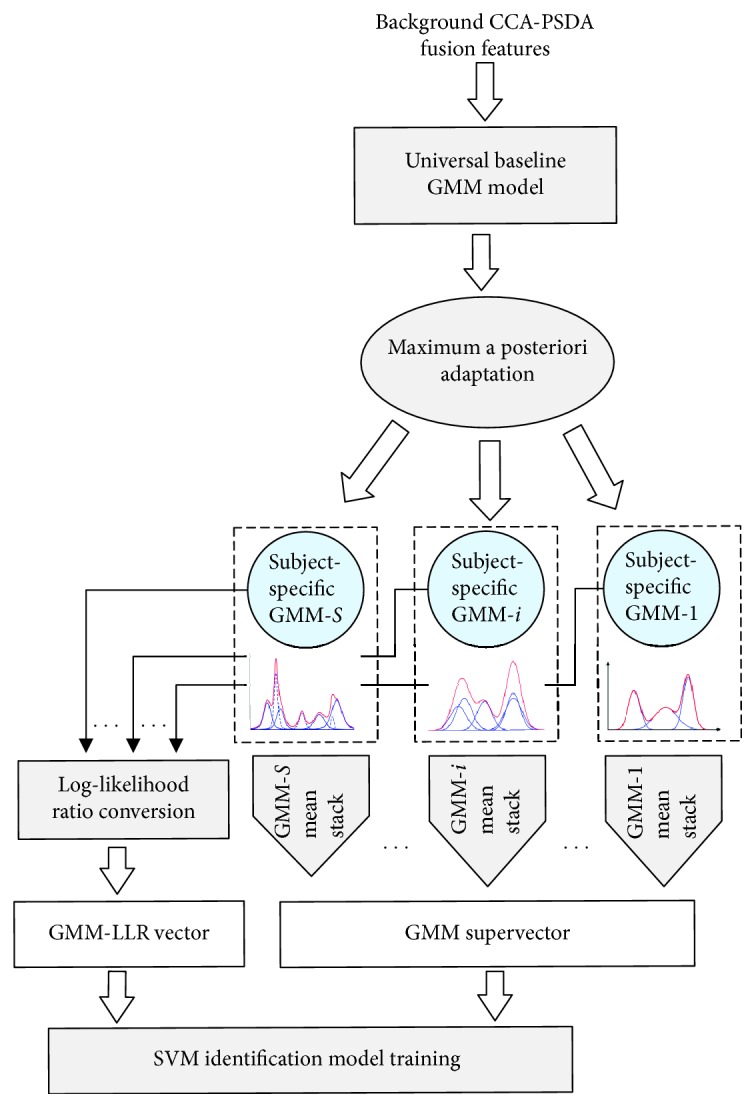
GMM-based discriminative likelihood vector and supervector transformation.

In order to better capture the subject-specific information obtained by the GMM-based model training and adaptation, we combine the generative GMM-based modeling with a support vector machine- (SVM-) based discriminative analysis as illustrated in Figure 9. After performing GMM training and adaptation, the subject-specific GMMs (i.e., MAP-adapted GMMs using target subject SSVEP segments) can generate scores for the input data. Hence, we can consider the set of subject-specific GMMs as a discriminative feature space transformation. In this way, the GMM likelihood scores were converted into log-likelihood ratios (LLRs) [40]. Then GMM-LLR scores were concatenated into a transformed vector and were fed to a predictive model for training and validation. However, to capture a higher resolution of the GMM information in the discriminative transformation, subject-specific GMM supervectors were also generated by extracting, stacking, and concatenating the MAP-adapted mean values of the Gaussian mixtures from each of the subject-adapted GMMs. The supervector transformation potentially generated a high-dimensional space. Finally, support vector machine (SVM) discriminative model training, which is a discriminative classifier that is robust to high dimensionality, was employed for classification. SVM complexity is dependent on the number of support vectors rather than the number of the input space dimensions.

### 4.7. Support Vector Machine (SVM)

To perform predictive modeling and validation, SVM was employed as a classifier in this study. SVM has been investigated in numerous and various applications and is well known for its ability to provide an efficient classification strategy to divide the input vectors into a 2-class problem. Additionally, SVM's ability to maximize the margin is attributed to a soft margin objective function that penalizes misclassified and within the margin samples as follows:

(6) min 12W2+C∑iξi yiw·xi+b≥1−ξi,∀xiξi≥0

as 2/‖*W*‖ represents the between classes margin and *ξ*_*i*_ denotes the degree to which a sample, *x*_*i*_, is within the margin so as to be penalized, whereas the soft margin algorithm seeks to maximize the margin while maintaining *ξ*_*i*_ at 0. However, it is worth mentioning that the algorithm does not decrease the number of misclassified samples, rather it minimizes the sum of the distances from the hyperplanes of the margin. Furthermore, the trade-off margin width and misclassification are controlled by the *C* coefficient.

SVM aims to project the input vector *x* into a scalar value *f*(*x*) to be the output score:

(7) $$\begin{array}{c} f\left(\;x\;\right)=\overset{N}{\underset{i=1}{\sum }}\alpha_{i}y_{i}k\left(\;x_{i},x\;\right)+b, \end{array}$$

where the support vectors are {*x*_*i*_∣*i* = 1,…, *N*}, the number of support vectors is *N*, the adjustable weights are *α*_*i*_ > 0, *y*_*i*_ = {−1, +1}, the bias term is *b*, and the kernel function is *K*(*x*_*i*_, *x*) = *ϕ*(*x*_*i*_)^*t*^*ϕ*(*x*), where *ϕ*(·) represents the mapping of the input space to a high-dimensional space. Moreover, the sign of *f*(*x*) determines the class decision for the 2-class classification problem. As such, we observe that sums of the kernel function construct the classifier as follows:

(8) $$\begin{array}{c} K\left(\;x_{i},x\;\right)=\phi {\left(\;x_{i}\;\right)}^{t}\phi \left(\;x\;\right), \end{array}$$

where *ϕ*(*x*) represents a mapping of the input space to a potentially infinite-dimensional space. The kernel of the radial basis function (RBF kernel), also known as a Gaussian kernel, is formulated as radial basis function (i.e., Gaussian function):

(9) $$\begin{array}{c} k_{RBF}\left(\;x,x^{\prime }\;\right)=\exp\left(\;-\frac{{\left\|x-x^{\prime }\right\|}^{2}}{2\sigma^{2}}\;\right), \end{array}$$

where *x* and *x*′ denote 2 samples that represent feature vectors in a certain input space. The squared Euclidean distance between both feature vectors is denoted by ‖*x* − *x*′‖^2^, while *σ* represents a free parameter.

## 5. Experimental Results

Data collection from 10 subjects was accomplished based on the procedure described in Section 4.1. Despite the inherent inaccuracies in the SSVEP stimulation, the data collection using the portable BCI4ICU system was accomplished with no loss of subject data.

In order to evaluate the SSVEP identification performance of the predictive model, 10-fold Cross-Validation (10 CV) was used. This entails partitioning the labeled training data into 10 equal-size subsets. Subsequently, 9 subsets are used to train the predictive model while the remaining subset is kept for validation to test the model's generalization capabilities on unseen data. This process runs 10 times while ensuring each subset is employed once as the validation subset. As such, the model's generalization capability is estimated by averaging the validation results. Then, SVM parameters are optimized utilizing simple grid optimization.

In this section, GMM parameter tuning, GMM-based subject identification, GMM-based SSVEP identification, and the information transfer rate will be discussed.

### 5.1. GMM Parameter Tuning

**Figure 10:**
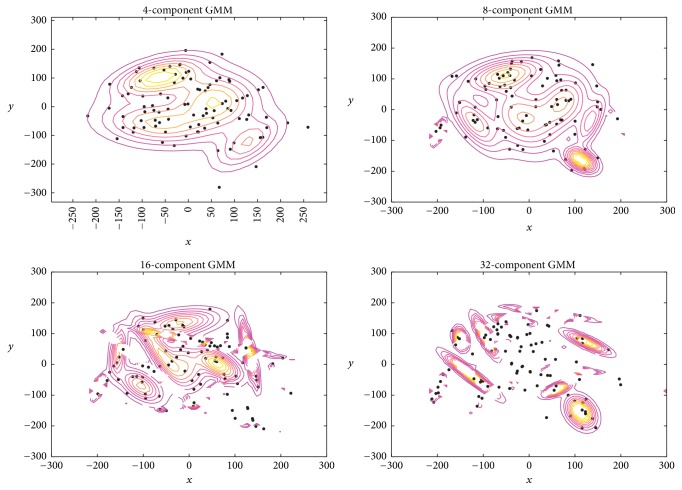
GMMs with different number of mixture components.

The number of mixtures is a very important parameter that requires close inspection and tuning. Different numbers of mixtures were selected for further observation.  Figure 10 depicts fitting a GMM employing 4, 8, 16, and 32 mixtures. From Figure 10, we note that GMM's population of samples is supported by 4, 8, and 16 mixtures. Conversely, estimated mean and variance of the training data did not support fitting a GMM with 32 mixtures due to the paucity of data samples. As such, only the first 3 numbers of mixtures (i.e., 4, 8, and 16) were employed for this investigation. Moreover, to obtain the optimal number of mixtures suited for our task and data, 10-CV SSVEP identification is conducted and the comparison is drawn accordingly.

### 5.2. GMM-Based Subject-Identification Results

To integrate the subject-specific information in the training phase of the SSVEP identification model, GMM training and adaptation are employed. In order to discover the level of subject-specific information present in the SSVEP data, we first conducted a subject-identification investigation using ARGMAX operator on GMM scores. We then generated 10-CV subject-identification accuracies of the subject-specific GMMs.  Table 2 reports the results employing (1) training GMMs from scratch on each subject's SSVEP data and (2) MAP-adapted GMMs, where a UBM was fitted to background data of all subjects and subsequently generated the subject-specific GMMs to adapt to each individual subject utilizing their SSVEP data.

From Table 2 we observe that the accuracy of identifying subjects using their SSVEP data with different numbers of mixtures was higher for the GMM-MAP approach as opposed to training GMMs from scratch. As such, the calibration time can be significantly minimized using GMM-MAP adaptation. Furthermore, GMM-MAP achieved an 87.3% accuracy when utilizing 8 mixtures. However, we observe that the identification performance of the GMMs trained from scratch exacerbates due to the lack of the data samples required to estimate the higher number of GMM parameters from scratch.

### 5.3. GMM-Based SSVEP Identification Results

Following the generation of the subject-specific scores for the MAP-adapted GMMs, we performed the GMM-based discriminative transformation of CCA-PSDA fusion features and conducted a 10-CV identification evaluation of the trained SVM predictive model (i.e., the procedure illustrated in Figure 9). The GMM scores were concatenated to generate a 10-dimensional transformed vector and then passed to the SVM predictive model (i.e., GMM-MAP-SVM). Also, the adapted mean scores of each of the subject-adapted GMMs were stacked and concatenated to generate the GMM mean super vectors. Given the 3 number of mixtures employed (i.e., 4, 8, and 16), the supervectors were 40-dimensional, 80-dimensional, and 160-dimensional vector spaces (i.e., GMM-MAP-SSVM). The 10-CV SSVEP identification results using GMM-MAP-SVM and GMM-MAP-SSVM utilizing the 3 numbers of mixtures are reported in Table 3.

From Table 3, we note that GMM mean supervectors of all mixture means achieved considerable improvement compared to GMM-LLR score vectors. GMM-MAP-SSVM achieved a 98.7% average identification accuracy using 8 mixtures compared to GMM-MAP-SVM which achieved a 92.91% average accuracy with the same number of mixtures. That entails greater than 5% relative improvement. As such, the experimental results demonstrate that our proposed closed-loop and portable BCI4ICU system is robust and can be utilized for effective communication for patients in the intensive care.

### 5.4. Information Transfer Rate (ITR)

All the results in this paper stem from offline data analysis. As such, we follow Meinicke et al. [41] and compute the information transfer rate as follows:

(10) Bt=t60log2⁡M+Plog2⁡P+1−Plog2⁡1−P/M−1,

where *B*_*t*_ represents the ITR in bits/min, *t* indicates the required time for each trial, *M* represents the number of target frequencies displayed on the visual stimuli, and *P* refers to the probability that the desired icon will be selected (i.e., accuracy).

Table 4 reports the information transfer rates of our portable BCI system across all 10 subjects. The baseline column represents our system's performance, which demonstrates an average ITR of 17.73% bit/min, whereas GMM-MAP-SVM improved the ITR to 25.94%. Finally, GMM-MAP-SSVM further enhanced the average overall ITR across all 10 subjects to 27.49% and GMM-MAP SSVM demonstrated the information transfer rates achieved by our proposed method.

Several efforts investigated the information transfer rates of BCI systems. Reagor et al. examined maximizing the ITR of SSVEP-based BCIs utilizing a tablet interface design [42]. Their experimental results on 5 subjects demonstrate that their overall accuracy and ITR without giving user feedback were 94.75% and 32.66 bit/min, respectively. However, when providing user feedback their overall accuracy and ITR were 96.34% and 27.56 bit/min. Furthermore, the majority of such efforts utilize cathode ray tube (CRT) and/or computer monitors with relatively high screen refresh rates. Yuan et al. investigated estimating the ITR of various EEG amplifiers utilizing 3 different paradigms, P300, motion, and SSVEP [43]. Their findings illustrate that the highest SSVEP accuracy and ITR achieved were 80.49% and 24.5 bit/min, respectively.

## 6. Conclusion

In this work, we attempted to address the needs of patients in the intensive care by developing a rapid and effective communication system that utilizes an SSVEP-based BCI, a wearable EEG cap, and an Android tablet to serve as the visual stimuli. We proposed a novel subject-specific GMM-based training and adaptation where we integrated the subject-specific information into the training of the SSVEP identification model, obtained subject-identification accuracies from the subject-specific GMMs, and finally generated the transformed vectors which are then passed to the predictive model. Our experimental results demonstrated that the GMM-MAP-SVM achieved 92.91% with 8 mixtures, while the GMM-MAP-SSVM was more robust achieving 98.7% identification accuracy also with 8 mixtures. Hence, the proposed system can be employed for effective and consistent communication in an ICU environment.

After this successful validation on our population of 10 subjects, we intend to perform a bench-to-bedside pilot study on mechanically ventilated patients in the ICU, with a user interface designed with input from intensive care physicians, critical care nurses, and speech/language pathologists.

## Figures and Tables

**Table 1:** Frequencies conversion values from Hertz to milliseconds versus our systems performance over 4 epochs.

Target frequencies	Hz to ms	1st epoch	2nd epoch	3rd epoch	4th epoch	Average
8.5 Hz	117.647	115.7391	116.913	116.6957	113.2083	115.639
10 Hz	100	98.92308	100.1923	100.1154	100.1538	99.84615
12 Hz	83.3333	82.125	83.5	83.40625	83.40625	83.10938
15 Hz	66.6666	65.71795	66.79487	66.69231	65.95	66.28878

**Table 2:** GMM-based 10-CV subject identification accuracies.

	4 mixtures	8 mixtures	16 mixtures
GMM-MAX	80.3	78.5	72.8
GMM-MAP-MAX	82.6	87.3	85.2

**Table 3:** Our system's performance versus GMM-MAP-SVM and GMM-MAP-SSVM identification accuracies using 3 different numbers of mixtures.

Subjects	CCA	PSDA	GMM-MAP-SVM			GMM-MAP-SSVM		
			4 mixtures	8 mixtures	16 mixtures	4 mixtures	8 mixtures	16 mixtures
Subject 1	83%	67%	92.00%	95.30%	94.40%	98.30%	100.00%	100.00%
Subject 2	77%	83%	93.60%	96.70%	92.10%	96.50%	98.70%	93.20%
Subject 3	40%	26%	91.70%	93.60%	90.60%	90.80%	98.40%	94.90%
Subject 4	59%	19%	89.40%	90.20%	89.70%	87.20%	97.70%	94.20%
Subject 5	67%	41%	92.30%	93.90%	93.80%	93.70%	98.80%	97.60%
Subject 6	55%	35%	90.00%	91.50%	92.50%	92.00%	96.90%	96.60%
Subject 7	62%	29%	90.20%	90.60%	93.80%	96.60%	100.00%	99.70%
Subject 8	71%	39%	86.50%	89.50%	88.70%	87.80%	98.50%	96.50%
Subject 9	47%	17%	89.80%	92.00%	89.30%	92.70%	99.20%	95.80%
Subject 10	61%	17%	87.70%	95.80%	91.90%	95.40%	98.80%	96.30%

*Average *	*62%*	*37%*	*90.32%*	*92.91%*	*91.60%*	*93.10%*	*98.70%*	*96.40%*

**Table 4:** Information transfer rates of our system and the 8-mixture model for both GMM-MAP SVM and GMM-MAP-SSVM.

Subjects	Baseline	GMM-MAP-SVM	GMM-MAP-SSVM
Subject 1	23.30%	26.58%	27.83%
Subject 2	21.69%	26.95%	27.49%
Subject 3	11.76%	26.13%	27.41%
Subject 4	16.87%	25.22%	27.22%
Subject 5	19.02%	26.21%	27.51%
Subject 6	15.80%	25.56%	27%
Subject 7	17.68%	25.32%	27.83%
Subject 8	20.09%	25.03%	27.43%
Subject 9	13.65%	25.70%	27.62%
Subject 10	17.41%	26.71%	27.51%

*Average *	* 17.73% *	* 25.94% *	* 27.49% *
